# Transplants in Brazil: where are we?

**DOI:** 10.6061/clinics/2019/e832

**Published:** 2019-05-13

**Authors:** Paulo Manuel Pêgo-Fernandes, José Osmar Medina Pestana, Valter Duro Garcia

**Affiliations:** I Cardiopneumologia, Faculdade de Medicina FMUSP, Universidade de Sao Paulo, Sao Paulo, SP, BR; II Nefrologia, Universidade Federal de Sao Paulo (UNIFESP), Sao Paulo, SP, BR; III Complexo Hospitalar Santa Casa de Porto Alegre, Porto Alegre, RS, BR

The modern era of transplantation began in the early 1900s when surgeons Aléxis Carrel and Charles C. Guthrie developed the blood vessel suture technique and performed experimental transplants. The first successful procedure was a kidney transplant performed in 1954 involving two twin siblings in Boston.

Transplantations in Brazil began in 1964, also starting with kidney transplants, followed by heart, liver, intestine and pancreas transplants, all of which first occurred in 1968. However, the results were discouraging, and the solid organ transplantation program, except for kidneys, was suspended at a global level.

The entire process of organ transplantation was nascent, as were the structure of Intensive Care Units and the techniques used for preserving organs outside the body. Elucidating the relevant immunological concepts was even more critical for the success of the transplants because little was known about rejection and there were few drugs capable of preventing it.

With the discovery of a new immunosuppressant (cyclosporine) in the 1970s, the development of an effective preservation solution and the standardization of organ removal, transplantation procedures obtained encouraging results starting in the 1980s (1).

Our country has pioneered numerous advancements in this field. Just a few examples of the innovative Brazilian spirit are Professor Euryclides de Jesus Zerbini, who performed the first heart transplant in Latin America (and the sixth in the world) in 1968; Prof. Silvano Raia, who performed the first liver transplant in the world; and Prof. José Medina who coordinates the world's largest kidney transplant program.

Currently, Brazil ranks second among all countries with regard to the number of transplants performed and has the highest level of public funding for this procedure; 95% of transplants in the country are funded by the SUS (public health care system).

Over the past seven years, the effective donor rate has grown 69%, going from 9.9 donors per million population (pmp) to 16.7 donors pmp; the reporting rate of potential donors has increased 41%, and donation effectiveness has risen by 21%.

In 2017, 5,929 kidney transplants (28.8 pmp) were performed, which was 41.1% of the 14,425 transplants needed (70 pmp). The number of transplants with cadaver donors has been increasing and the number of living donors has decreased, probably due to caution regarding the risk of late donor complications.

Liver transplants have increased by 12% to 2,109 transplants (10.2 pmp), which account for only 40.9% of the 6,182 transplants required (30 pmp), and São Paulo was the state that performed the largest number of procedures in 2017.

In 2017, 380 heart transplants were performed (1.8 pmp), which was 23% of the 1,649 transplants needed (8 pmp); it is important to note that the agency's utilization rate has increased by 11%, but much remains to be done to reach the established target of 40%.

Lung transplants were performed in only 4 states in 2017, with a total of 112 procedures (0.5 pmp), accounting for only 6.7% of the 1649 transplants required (2 pmp); the 3.2% utilization rate is still far below the required 20%. One of the main reasons for this low utilization rate is the small number of working teams (2).

For the transplant system to advance, it is vital that the following components of the donation process improve: legislation, funding, organization and education. Necessary legal measures include implementing a voluntary donor registration system and gaining greater control over transplantation among nonfamily donors.

Regarding the financial measures, it has been 11 years since the compensation for the hospitals and transplantation teams was readjusted. Other procedures, such as posttransplant follow-up, have received the same reimbursement for 20 years.

We need a supportive overview from the National Private Healthcare System Agency (Agência Nacional da Saúde Suplementar – ANS) and private healthcare companies to give patients the support for transplantation coverage, if needed. This improvement in funding is critical to ensure the sustainability of the system.

Together with the support of the press, we must deepen the population's knowledge of the transplant system to continue to encourage organ donation. Organ capture logistics also pose challenges that need to be addressed, given the vast size of our country. Coverage requires the use of several types of transportation for safe and fast pickups in increasingly distant locations. The motivation of medical teams working in critical units for the diagnosis of brain death and adequate maintenance of potential donors is essential.
